# Relationship Between the Number of Occlusal Supporting and Medical Cost: Analysis Using Large Claims Database from Employee Health Care Insurance in Japan

**DOI:** 10.36469/001c.11594

**Published:** 2020-01-24

**Authors:** Tatsunori Murata, Korenori Arai, Kosuke Kashiwagi, Shunsuke Baba

**Affiliations:** 1 Department of Oral Implantology, Osaka Dental University, Hirakata Japan; 2 Department of Fixed Prosthodontics and Occlusion, Osaka Dental University, Hirakata Japan

**Keywords:** occlusal support, medical expenditure, claims database, Eichner classification, Miyachi classification

## Abstract

**Background:**

There are several previous reports suggesting that the number of remaining teeth is related to increase of total medical expenditure. However, the measurements of oral healthcare conditions used in the previous studies were the number of remaining teeth, and occlusal support was not assessed.

**Objectives:**

The aim of this study was to evaluate the relationships between occlusal support and healthcare resource utilization.

**Methods:**

This study was a retrospective cohort study using a claims database. Measurements of occlusal support were defined by the Eichner and Miyachi classification systems based on dental formula information. Medical healthcare resource usage was measured by medical visit rate and 12-month medical expenditure.

**Results:**

Of the total population in the claims database, 1 288 713 patients were included in the analysis. The proportion of patients who had at least one medical visit and annual medical expenditure in the best condition classes in each classification measure (i.e. A1 for Eichner classification and Area I for Miyachi classification in both endpoints) were 58.2% and 61.1%, and JPY34 597 (US$314.52 at JPY110/US$) and JPY43 129 (US$392.08), respectively. Those in the poorest condition classes in each classification measure (i.e. B3 for Eichner classification and Area IV for Miyachi classification in the medical visit rate, and C1 for Eichner classification and Area III for Miyachi classification in medical expenditure) were 75.3% and 75.1%, and JPY149 339 (US$1357.63) and JPY120 925 (US$1099.32), respectively. We found a positive correlation with the outcomes by regression analysis adjusting for deterioration of occlusal support with age and gender.

**Conclusion:**

We found significant relationships between occlusal support conditions and healthcare resource utilization. The maintenance of oral health or dental treatment may positively impact overall health, and active dental intervention may reduce the total medical expenditure.

## INTRODUCTION

The national healthcare expenditure in Japan has continuously increased due to the aging of society and the introduction of high-cost technologies. The annual national healthcare expenditure was JPY4.2 billion (US$38 million at the exchange rate of JPY110 per US$), with 70% being for medical treatment (JPY3.0 billion, US$27 million) and 7% for dental treatment (JPY0.3 billion, US$2.7 million). The difference between medical and dental expenditure was significant.^1^

Several previous studies evaluated relationships between oral healthcare and the overall healthcare condition and suggested that the oral condition is related to survival, mortality from cardiovascular disease, and risk of dementia.^2^–^4^ Furthermore, there are several reports suggesting that the number of remaining teeth is related to total medical expenditure.^5^–^11^ Studies in several several regions in Japan, including Hyogo, Ibaraki, Hokkaido, Yamanashi, and Nagano Prefectures, reported that the medical expenditure by patients with ≤4 teeth was 1.4- or 1.6-times that of patients with ≥20 teeth.^5^–^10^ In the study by Tsuneishi et al. using the largest claims database called the National Data Base, which covers approximately 99% of the population of Japan, the medical expenditure by patients with ≤19 teeth was significantly higher than that by patients with ≥20 teeth based on analysis of a population of 2.2 million.^11^ However, the measurements of oral healthcare conditions used in the previous studies were mainly the number of remaining teeth, and occlusal support was not assessed. The reductions of occlusal support were related to worsening of patient’s mastication, periodontal protection and quality of life^12^ and also expected to have a potential impact on overall healthcare condition. Therefore, the aim of this study was to evaluate the relationships between occlusal support conditions and healthcare resource utilization including medical expenditure data.

## MATERIALS AND METHODS

### Study design and data source

This study was a retrospective cohort study using a medical claims database provided by Japan Medical Data Center Co. Ltd (JMDC database). The study obtained the approval (110946) of the Ethics Committee of Osaka Dental University.

The JMDC database consists of data concerning claims for hospitalization, outpatient visits, dispensation, and physical examinations provided by health insurance societies. As of December 2018, the database stores clinical information from January 2005 and anonymized data of a cohort consisting of a total of approximately 8 million people belonging to more than 90 health insurance societies. As the JMDC database can be identified using IDs given to individual subscribers by health insurance societies, patients can be traced even when they have been transferred to other hospitals or are treated at multiple institutes.

### Study population

The study period was from April 2016 to March 2017. Patients who fulfilled the following two selection criteria were included: (1) a continuous subscriber for at least 12 months during the study period, (2) had a record of definitive diagnosis of gingivitis or periodontal disease (defined as ICD10 code: K05) during the study period, and (3) were aged over 20 years as of March 2017.

### Variables and endpoints

**Figure 1:**
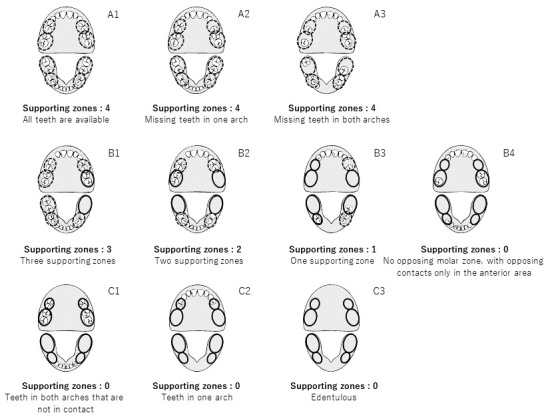
Definition of occlusal support conditions by the Eichner classification Of the ten scores of the Eichner classification, “Al” has all contacts, “A2” has missing teeth in one arch, and “A3” has missing teeth in both arches. “Bl” has three support zones, “B2” has two support zones, “B3” has one support zone, and “B4” has no opposing molar zone, with opposing contacts only in the anterior area. “Cl” scores have teeth in both arches that are not in contact; “C2” scores indicate teeth in one arch, whereas “C3” indicates that the subject is edentulous.

Measurements of occlusal support was defined by the Eichner classification and Miyachi classification systems based on dental formula information from the claims data in the study period. The Eichner classification was developed by Eichner and defined by the conditions of occlusal support shown in Figure 1.^13^ In the Eichner classification, each posterior contact area (premolar and molar) is counted as one region, for a total of four support zones. All “A” scores refer to occlusal support in all four premolar and molar regions; “Al” has all occlusal support, “A2” has missing teeth in one arch, and “A3” has missing teeth in both arches. All “B” scores refer to occlusal support in 0–3 posterior regions; “Bl” has three support zones, “B2” has two support zones, “B3” has one support zone, and “B4” has no opposing molar zone, with opposing support only in the anterior area. No “C” scores have opposing support; “Cl” scores have teeth in both arches that do not function in occlusal support; “C2” scores indicate teeth in one arch, whereas “C3” indicates that the subject is edentulous.

**Figure 2:**
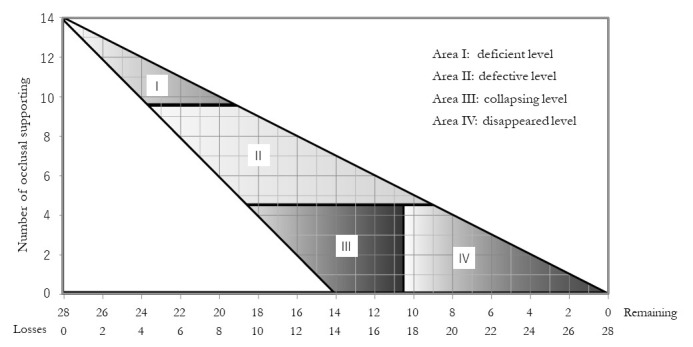
Definition of occlusal support conditions by the Miyachi classification Of the four scores of the Miyachi classification, Area I indicates a deficient level with ≥10 remaining occlusal support points and 1 to 8 missing teeth. Area II indicates a defective level with 5 to 9 remaining occlusal support points and 5 to 15 missing teeth. Area III indicates a collapsing level with ≤4 remaining occlusal support points and 10 to 18 missing teeth. Area IV indicates disappearance with ≤4 remaining occlusal support points and ≥18 missing teeth.

The Miyachi classification was proposed by Miyachi et al. There are four categories defined by the number of occlusal support points and remaining teeth, as shown in Figure 2.^14^ Area I indicates a deficient level with ≥10 remaining occlusal support points and 1 to 8 missing teeth. Area II indicates a defective level with 5 to 9 remaining occlusal support points and 5 to 15 missing teeth. The appearance of non-vertical stop occlusion is possible. Area III indicates a collapsing level with ≤4 remaining occlusal support points and 10 to 18 missing teeth. The risk of non-vertical stop occlusion is significantly increased and proactive intervention is required. Area IV indicates disappearance with ≤4 remaining occlusal support points and ≥18 missing teeth. The risk of non-vertical stop occlusion is reduced and the oral condition is stable.

The poorest classifications for each measurement in the study period were used to analyze the relationship between occlusal support conditions and healthcare resource utilization in medical fields.

Healthcare resource utilization in medical fields was measured by the medical visit rate, including both outpatient and inpatient visits (i.e. the proportion of patients who had at least one medical visit during the study period), and 12-month medical expenditure for patients who had at least one medical visit during the study period.

### Statistical analyses

To clarify the non-adjusted relationships between occlusal support conditions and healthcare resource usage, crude stratified analyses by the Eichner classification and Miyachi classification were carried out for the medical visit rate and 12-month medical expenditure for patients who had at least one medical visit during the study period.

Furthermore, as age and gender independently affect the variables and endpoints, in order to evaluate age- and gender-adjusted relationships between occlusal support conditions and healthcare resource usage, we performed regression analyses using the logistic regression model and gamma regression model for the medical visit rate and medical expenditure, respectively. Point estimates and 95% confidence intervals for the odds ratio and exponential of regression coefficients, respectively, were assessed to interpret the magnitude of impact of the occlusal support condition on the endpoints and its significance.

## RESULTS

### Patient characteristics

**Figure 3:**
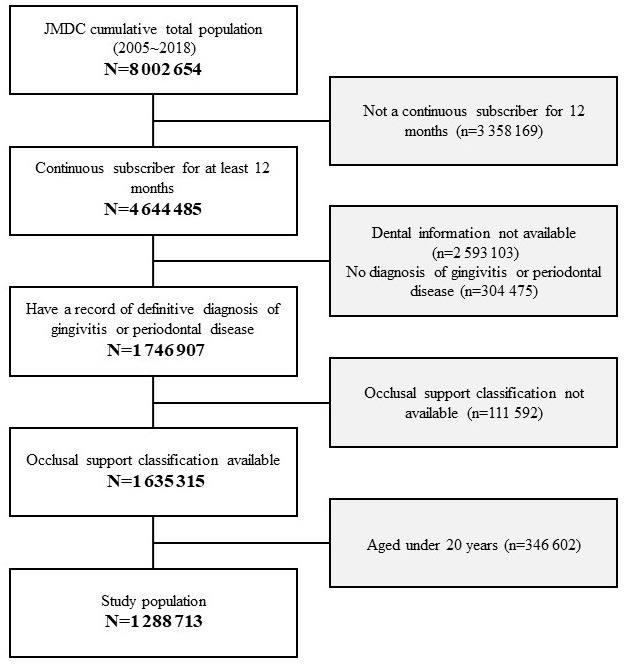
Patient selection flow chart Of the total population in the JMDC claims database, 1 288 713 patients were included in the analysis, excluding those with missing information for occlusal support classification from 4 644 485 patients who fulfilled the selection criteria.

Of the total population that was available in the JMDC claims database, 1 288 713 patients were included in the analysis, excluding those with missing information for occlusal support classification who fulfilled the selection criteria (Figure 3). The mean age of the study population was 44.8 years (standard deviation: 12.4 years) and 48.2% of the patients were female (Table 1). The medical visit rates were independently related with both gender and age, but annual medical expenditures for patients who had at least one medical visit were increased only by age (Table 2).

### Relationship between occlusal support conditions and healthcare resource utilization

The results of crude stratified analyses by occlusal support conditions are shown in Table 3. The medical visit rate and annual medical expenditure in the best condition classes in each classification system (i.e. A1 for Eichner classification and Area I for Miyachi classification for both endpoints) were 58.2% and 61.1%, and JPY34 597 (US$314.52 at the exchange rate of JPY110 per US$) and JPY43 129 (US$392.08), respectively. Those in the poorest condition classes in each classification system (i.e. B3 for Eichner classification and Area IV for Miyachi classification in the medical visit rate, and C1 for Eichner classification and Area III for Miyachi classification in medical expenditure) were 75.3% and 75.1%, and JPY149 339 (US$1357.63) and JPY120 925 (US$1099.32), respectively. The impact on the endpoints by the occlusal support condition was consistent between the classification systems.

**Figure 4:**
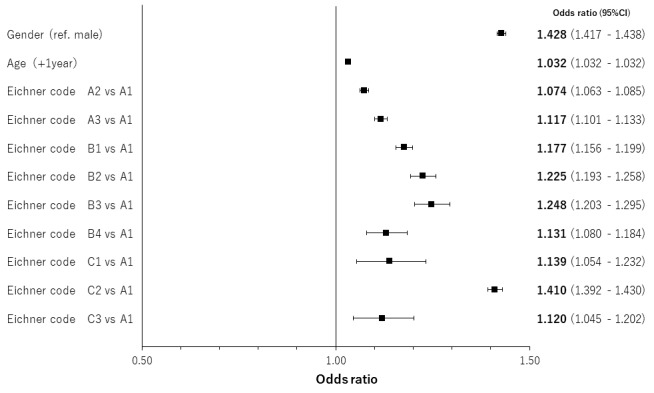
Age- and gender-adjusted relationships between the Eichner classification and the medical visit rate CI: confidence interval

**Figure 5:**
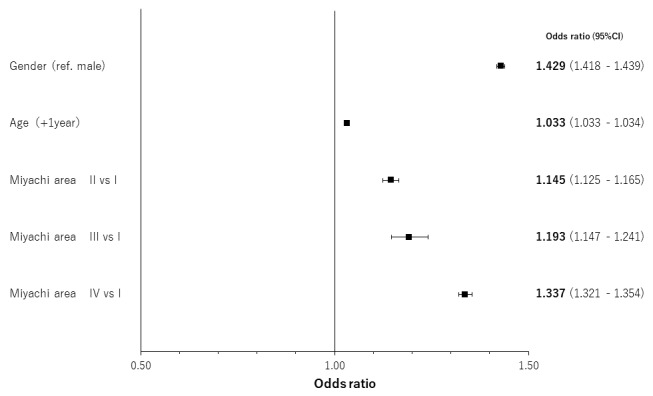
Age- and gender-adjusted relationships between the Miyachi classification and the medical visit rate CI: confidence interval

**Figure 6:**
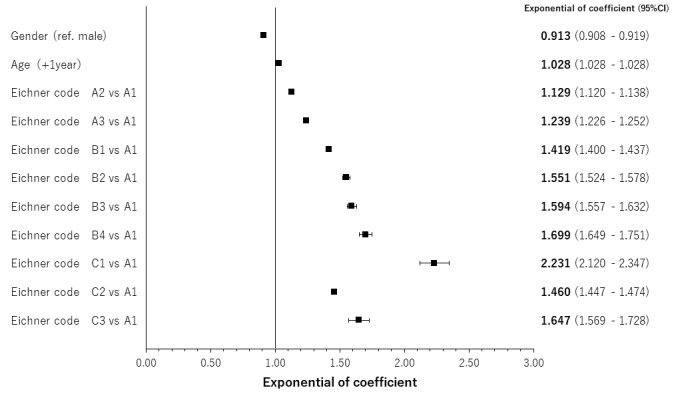
Age- and gender-adjusted relationships between the Eichner classification and medical expenditure CI: confidence interval

**Figure 7:**
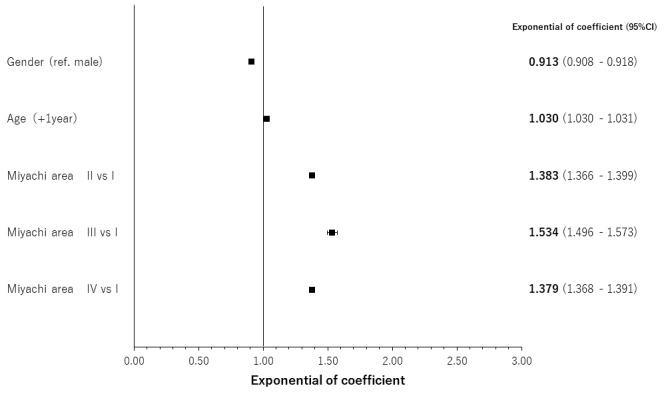
Age- and gender-adjusted relationships between the Eichner classification and medical expenditure CI: confidence interval

The results of regression analyses by occlusal support conditions for age- and gender-adjusted relationships are shown in Figures 4 to 7 by both classification systems and for both endpoints. We found a positive linear correlation between the medical visit rate and medical expenditure, and deterioration of occlusal support conditions. In the poorest condition classes for the medical visit rate in the Eichner classification (i.e. C2) and Miyachi classification (i.e. Area IV), the odds ratios of the medical visit rate against the best condition classes were 1.410 (95% confidence interval: 1.392 – 1.430) and 1.337 (95% confidence interval: 1.321 – 1.354), respectively. In the poorest condition classes for medical expenditure in the Eichner classification (i.e. C1) and Miyachi classification i.e. (Area III), the exponential of the regression coefficient of medical expenditure against the best condition classes was 2.231 (95% confidence interval: 2.120 – 2.347) and 1.534 (95% confidence interval: 1.496 – 1.573), respectively. This suggests that medical expenditure by patients in the C1 Eichner classification category is 2.231-times higher than that by patients in the reference category of A1. However, we also found a negative correlation between healthcare resource utilization and the total or almost complete loss of occlusal support (e.g. C3 in the Eichner classification).

## DISCUSSION

The present study was performed to assess the relationships between occlusal support conditions and healthcare resource utilization. In the analysis based on the Eichner classification, there was a positive correlation between the medical visit rate and medical expenditure. Our results suggest that the deterioration of occlusal support conditions influences the overall healthcare condition of patients and increases the need of medical intervention. This interpretation can be supported by several previous studies reported that poor oral conditions are associated with lower intake of fruits and vegetables,^15^ and then with higher risks of cardiovascular disease and stroke,^16^ periodontal problems can cause chronic systemic inflammation,^17^ which are related to an increased prevalence of metabolic syndrome.^18^ Moreover, acceleration of active dental interventions to prevent or treat oral problems may be an effective political option to control the increased total healthcare expenditure.

The increases in healthcare resource utilization in the Miyachi classification were mild in comparison with those in the Eichner classification (the poorest exponential point estimates of coefficients were 1.534 in Area III of the Miyachi classification versus 2.231 in B2 of the Eichner classification for medical expenditure). One possible reason for this is that a broader patient population was used for the Miyachi classification because the range of covered patient characteristics in the Miyachi classification is wider and the possibility to include patients with non-severe occlusal support conditions was higher than those in the Eichner classification. Although we collected a sufficient sample size for each occlusal support condition category in the detailed Eichner classification, there may be cases in which the Miyachi classification is prioritized such as when small sample sizes are used. We also found that medical expenditure was lower in the poorest occlusal status patients (C for Eichner classification / IV for Miyachi classification) than in the next severe category (B / III, respectively). The findings would potentially suggest that if the number of remaining teeth are quite a few or nothing, prosthesis practice would be easy to control, risk of periodontal problem and overall healthcare worsening could be reduced.

Several previous studies assessed the relationship between oral health and medical expenditure similar to the present study.^5^–^11^ The results of these previous studies were consistent with our study and support that the deterioration of oral healthcare increases medical expenditure. Also, the study that performed in oversea country was also suggested the similar results. Kim examined the oral health conditions and oral health behaviors of current high-cost patients and to evaluate which oral health measures identify future high-cost patients using Korean national database. He demonstrated that oral health measures are associated with the risk of becoming a high-cost patient. The results highlight the impact of oral health on healthcare costs and he reached the same conclusion with us.^19^ However, they did not evaluate occlusal support conditions.

## LIMITATIONS

This study, which used the data of a claims database from health insurance societies, has some limitations.

The first limitation is the generalization. As the JMDC database collects information primarily concerning patients who are employed members of health insurance societies, the accumulated data are considered primarily those of patients in a relatively good condition of health, and data for older individuals are limited.

The second limitation is internal validity. As the dental formula information in the JMDC database does not reflect the conditions of prosthetic treatment, the patients recognized as those with loss of occlusal support in this study by the Eichner or Miyachi classification systems may have been treated by prosthetic intervention and his/her oral conditions may be higher than the categorization. We believe that this limitation led to an underestimation of the relationship between occlusal support conditions and healthcare resource utilization in this study.

## CONCLUSION

The present study assessed the relationships between occlusal support conditions and healthcare resource utilization, demonstrating significant relationships between them. The maintenance of oral health or dental treatment may positively affect the overall health condition, and active dental intervention may reduce the total medical expenditure. However, it is difficult to evaluate causal relationships because the data source of the study was a claims database. Further studies are needed to support our conclusions.

## Figures and Tables

**Table 1:** Patient background information

	N	Gender (female)		Age		
	
		n	%	Mean	Median	Standard deviation
**Total**	1 288 713	621 686	48.2	44.8	45.0	12.4

**Age category**						

20–29	169 523	77 464	45.7	24.8	25.0	2.93
30–39	269 661	136 797	50.7	34.8	35.0	2.86
40–49	367 693	188 766	51.3	44.5	45.0	2.83
50–59	312 017	145 084	46.5	54.2	54.0	2.81
60–69	153 125	65 269	42.6	63.3	63.0	2.59
≥70	16694	8306	49.8	72.0	72.0	1.51

**Eichner classification**						

A1	697 045	342 208	49.1	40.1	40.0	11.1
A2	218 918	103 497	47.3	48.0	49.0	10.9
A3	106 898	56 408	52.8	48.9	50.0	11.4
B1	65 970	29 365	44.5	54.8	56.0	9.4
B2	31 642	13 904	43.9	57.1	59.0	9.2
B3	16 230	7233	44.6	58.7	60.0	8.7
B4	9961	4501	45.2	59.0	61.0	10.1
C1	3367	1489	44.2	58.8	61.0	9.9
C2	134 732	61 656	45.8	50.0	51.0	11.9
C3	3950	1425	36.1	55.5	56.0	9.3

**Miyachi classification**						

Area I	1 057 833	517 697	48.9	43.1	43.0	11.9
Area II	72 694	32 584	44.8	56.6	58.0	9.3
Area III	13 925	5919	42.5	58.6	60.0	9.5
Area IV	144 261	65 486	45.4	50.5	52.0	11.9

**Table 2:** The medical visit rate and 12-month medical expenditure by gender and age

	N	Proportion of medical visits1*		12-month medical expenditure (JPY)		
	
		n	%	Mean	Median	95% CI
**Total**	1 288 713	814 028	63.2	52 207	9140	51 732–52 683

**Gender**						

Male	667 027	397 541	59.6	53 762	7710	53 036–54 487
Female	621 686	416 487	67.0	50 539	10 700	49 934–51 144

**Age category**						

20–29	169 523	84 831	50.0	20 965	730	20 092–21 837
30–39	269 661	152 938	56.7	27 089	5140	26 436–27 742
40–49	367 693	218 808	59.5	39 364	7100	38 658–40 071
50–59	312 017	219 571	70.4	71 764	16 330	70 582–72 946
60–69	153 125	122 954	80.3	108 667	31 930	106 639–110 696
≥70	16 694	14 926	89.4	174 677	62 475	166 697–182 656

JPY1 = US$0.0091

1*: the proportion of patients who had at least one medical visit during the study period

**Table 3:** The medical visit rate and 12-month medical expenditure by occlusal support conditions

	N	Medical visit rate1*		12-month medical expenditure (JPY)		

		n	%	Mean	Median	95% CI
**Total**	1 288 713	814 028	63.2	52 207	9140	51 732–52 683

**Eichner classification**						

A1	697 045	405 889	58.2	34 597	6030 34	104–35 089
A2	218 918	143 333	65.5	54 304	11 160	53 231–55 377
A3	106 898	71 973	67.3	63 348	12 600	61 538–65 158
B1	65 970	47 320	71.7	87 623	19 480	84 435–90 811
B2	31 642	23 389	73.9	105 412	24 080	100 215–110 609
B3	16 230	12 217	75.3	113 347	27 670	106 778––119 915
B4	9961	7326	73.6	120 998	26 400	111 351–130 645
C1	3367	2475	73.5	149 339	25 180	127 643–171 035
C2	134 732	97 317	72.2	84 837	18 875	82 976–86 698
C3	3950	2789	70.6	103 968	21840	91 551–116 385

**Miyachi classification**						

Area I	1 057 833	646 184	61.1	43 129	7670	42 673–43 586
Area II	72 694	53 215	73.2	99 829	22 815	96 734–102 923
Area III	13 925	10 452	75.1	120 925	27 430	112 451–129 398
Area IV	144 261	104 177	72.2	88 144	19 190	86 235–90 053

JPY1 = US$0.0091

1*: the proportion of patients who had at least one medical visit during the study period
